# Treatment of Compound Fracture of the Jaws

**Published:** 1905-04-15

**Authors:** T. L. Gilmer

**Affiliations:** Chicago


### TREATMENT OF COMPOUND FRACTURE OF THE JAWS.

BY DR. T. L. GILMER. OF CHICAGO.

An intoxicated man fell from a window on to a stone walk and was brought to the hospital in a comatose condition. The blood was oozing from the right ear with continuous hemorrhage from the nose and mouth, which was stopped after several hours by packing iodoform gauze into the nostrils and fractured alveolus. The patient was kept at rest for four days under antiseptic treatment of the local lesions, as a preparatory treatment. A correct diagnosis showed that there were five fractures of the lower jaw. On the left side at the angle and at the first bicuspid. On the right side one at the neck of the condyle, one at the angle and one at the cuspid tooth. The upper jaw was fractured through the median line and broken from its attachments above. The six anterior teeth of the upper jaw had been knocked out and lost, the other teeth were in place. In the lower jaw only the left lateral incisor was lost.

The parts of the upper jaw were restored to normal position and an impression taken with very soft modeling compound, from which a plaster cast was obtained. A vulcanite plate was made on this, with square tubes invested along the buccal sides of the masticating teeth to receive the strong wire arms to be used for bandaging over the head. Into the lower surface of this plate wire staples were vulcanized, to be used for securing the approximation of the segments of the lower jaw by means of wire ligatures. The several segments of the fractured lower jaw were now brought together and secured by means of silver wire ligatures passed through holes drilled through the bone. German silver ligatures were now securely attached to several firmly-fixed teeth of the lower jaw, to be subsequently used to bring the lower jaw to proper occlusion. The splint was then fitted to the upper teeth and the upper jaw segments forced into proper relations and held there by lacing the wire arms to a skull cap. The lower jaw was then brought up to an approximately proper occlusion and fastened to the splint by means of the German silver ligatures, previously attached to the lower teeth, and which were fastened to the staples vulcanized into the splint.

Primary union took place, without any unusual developments. There was never any unfavorable symptoms. The patient was discharged from the hospital in five weeks. The result was entirely satisfactory. There was a good occlusion of the teeth and no deformity.—*November International.*

